# Association between Potentially Inappropriate Medication (PIM) Use and Risk of Hospitalization in Older Adults: An Observational Study Based on Routine Data Comparing PIM Use with Use of PIM Alternatives

**DOI:** 10.1371/journal.pone.0146811

**Published:** 2016-02-03

**Authors:** Heinz G. Endres, Petra Kaufmann-Kolle, Valerie Steeb, Erik Bauer, Caroline Böttner, Petra Thürmann

**Affiliations:** 1 AQUA-Institute Goettingen, Goettingen, Germany; 2 Chair of Clinical Pharmacology, Faculty of Health, University Witten/Herdecke, Witten, Germany; INRCA, ITALY

## Abstract

**Objective:**

The safety of potentially inappropriate medications (PIMs) in elderly patients is still debated. Using the PRISCUS list, we examined the incident all-cause hospitalization risk associated with PIMs compared to PIM alternatives during the 180 days post individual first pharmacy dispensing (index date).

**Methods:**

Routine claims data from a German health insurer on 392,337 ambulatory patients aged ≥65 years, were used to estimate adjusted hazard ratios (HRs) for hospitalization associated with incident PIM use. Observation period was January 2009 –December 2010. Users of PIM alternatives, as defined by the PRISCUS list, were the reference group. Patients with PIM dispensing or hospital stay in a six month “washout” period (second half of 2008) were excluded. All potential confounders were determined in the half year before the individual index date.

**Results:**

In the total cohort 60.7% were female. Median age was 73 years. Of 79,041 incident PIM users, 58.4% had PIMs dispensed in one quarter of 2009 or 2010, 19.3% in two quarters, and 22.3% in three or more quarters. There were 126,535 hospitalizations during the observation period, and 47,470 of them occurred within 180 days post first dispensing. Multivariable Cox regression analysis revealed PIM use as a significant risk factor for hospitalization (HR 1.378; 95% CI 1.349–1.407) compared to use of PIM alternatives.

**Conclusions:**

PIM use compared to use of PIM alternatives is associated with an increased risk of all-cause hospitalization in the 180 days following individual index date. Future analyses comparing a single PIM with its corresponding alternative may help identify those PIMs responsible for this.

## Introduction

Pharmacotherapy in adults 65 years of age or older is different from such treatment in younger patients. Comorbidities, multiple comedications, potential interactions, patient preferences, and physiologic decline in all steps of pharmacokinetics (PK) and pharmacodynamics (PD) must be considered. The geriatric dosing axiom, “start low and go slow” is based upon this PK/PD concern given that age-related changes in PK/PD alter the risk-benefit ratio of drug treatment [1]. Thus, older patients are at higher risk for potentially inappropriate medication and/or dosing, which may be compounded by their need for extensive pharmacotherapy of multiple chronic conditions [2, 3]. Several explicit lists of potentially inappropriate medications (PIMs) have been introduced to assist clinicians in screening for PIMs, starting with the 1991 Beers list for nursing home residents in the USA, subsequently expanded and revised in 1997, 2003, and 2012 in order to update evidence and to improve clinical relevance [2, 4–8]. Another recently published PIM list is the German PRISCUS list, which is specifically adapted for the requirements of the German drug market. As with the Beers list, PIMs for the PRISCUS list were identified and categorized by an interdisciplinary expert panel with the Delphi method used as the consensus process [7]. The PRISCUS list provides information regarding drugs to avoid, to be used with caution under certain circumstances, therapeutic alternatives, special comorbidities to consider, and monitoring advice if a PIM must be used. Most recently, a PIM list for several European countries was published [9].

However, published data are still too inconsistent to suggest that the use of PIMs is, in fact, significantly associated with a higher risk for adverse drug events (ADEs) [10]. Hence, some authors continue to doubt that the consideration of PIM criteria results in any improved (functional) outcome in the elderly [11, 12]. This doubt is supported by an article that found relatively few (1.2%– 6.6%, depending upon the PIM list used) emergency hospitalizations for ADEs in older adults due to medications specifically considered inappropriate [13]. Surprisingly, another study found a difference between PIMs defined by Beers criteria and PIMs defined by STOPP (Screening Tool of Older Persons' potentially inappropriate Prescriptions) criteria, with STOPP PIMs, but not PIMs from the Beers list, being significantly associated with avoidable hospitalizations in older people [14]. A systematic review of 18 studies that examined healthcare outcomes associated with PIMs (using Beers criteria) found that for hospitalization (8 of the 18 studies) the results were inconclusive as only half of the eight studies reported a significant association between PIM use and increased subsequent hospitalization [10]. In contrast, some very recent studies applying different methodological approaches and different PIM lists showed a significantly increased risk (between 13% and 63%) for hospitalizations associated with PIM use [15–17].

Since randomized controlled trials for PIM use are unethical, pharmacoepidemiologic studies that use large claim databases are the only alternative option available. Given that 22–25% of all patients aged ≥65 years in Germany are estimated to be PIM users [18, 19] we aimed to examine the global association between PIMs and hospitalizations in this age group. We focused on patients belonging to the largest statutory health insurer in southwestern Germany (AOK Baden-Württemberg), which covers about 40% of the total population (10.8 million people) in this federal state.

## Materials and Methods

We conducted a prospective cohort study based on routine data of practitioners of all medical specialties using claims data from AOK Baden-Württemberg. We deliberately chose the observation period January 2009 to December 2010 in order to avoid any influence on prescriptions by the publication of the PRISCUS list (in the second half of 2010). As an appropriate control group for patients exposed to a PIM (cases) we chose patients who received a medication classified as a safer PIM alternative in the PRISCUS list [7]. PIM alternatives belong to the same drug class or subclass used to treat the same conditions as treated by the PIMs. Since the data provides the quantity of active ingredient(s) per pill, we were able to detect dose-dependent PIM drugs, i.e. drugs that are only characterized as PIMs above a minimum dosage (e.g. Lorazepam > 2 mg/d).

**Fig 1:**
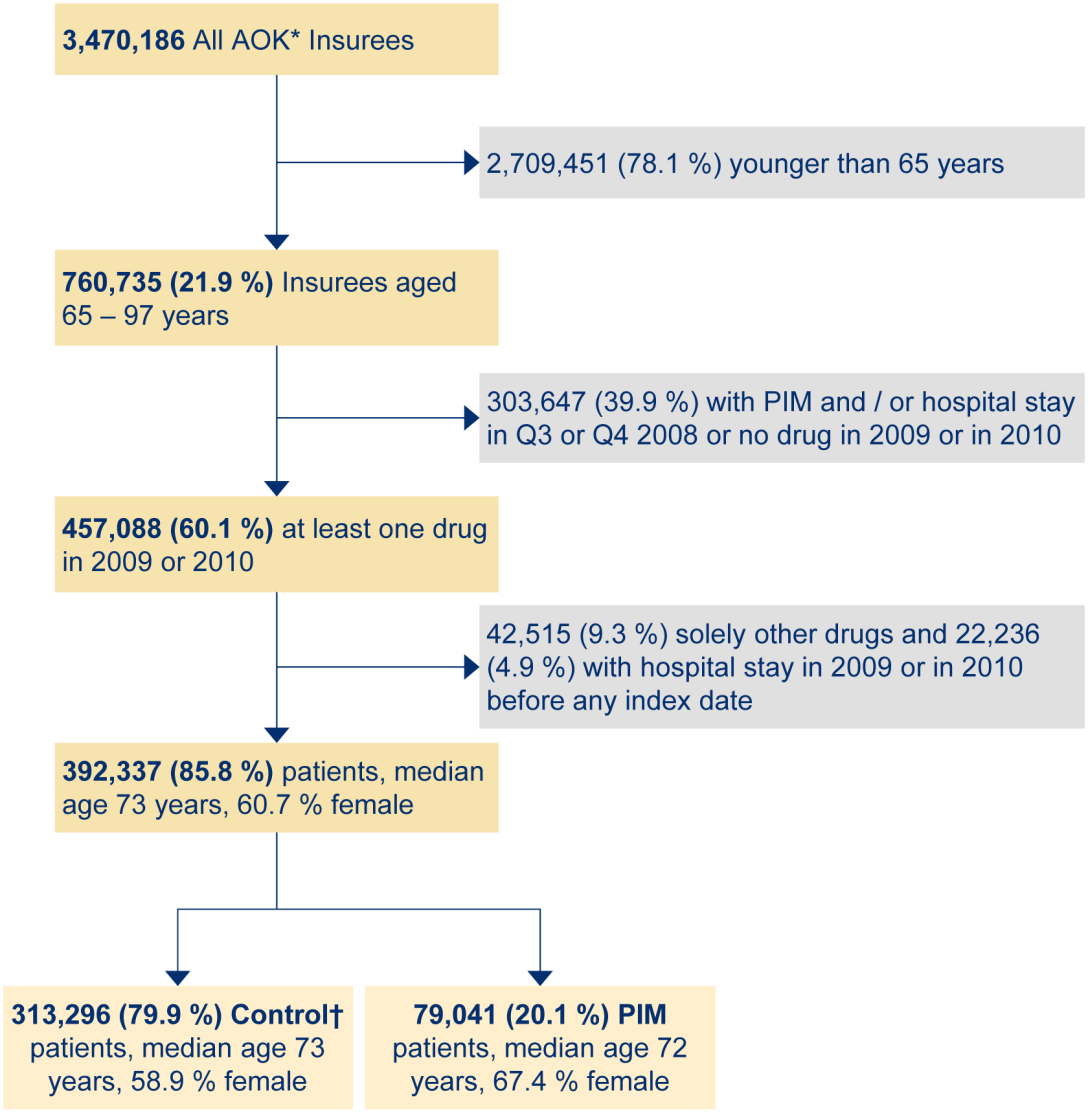
Study cohort selection. * Statutory health insurance company; † Control group with PIM alternatives as stated in the PRISCUS publication. Patients receiving solely “other” drugs were prescribed neither a PIM nor a PIM alternative.

Fig 1 is a flow chart that summarizes schematically the selection of this study cohort. In brief: After exclusion of implausible data and patients aged less than 65 years from the total data set, 21.9% of the 3.470 million insured people remained. In order to provide at least a half-year “washout” period for both, PIM use and hospitalizations, all patients with PIM use or hospitalization between July 1, 2008 and December 31, 2008 were excluded. We further excluded all persons with no drug claim in 2009 or in 2010 thus ensuring at least a minimum of drug exposure. Finally, we excluded persons who received no PIM alternatives in 2009 and/or in 2010 thus ensuring sufficient patients for the control group.

PIM and PIM alternatives in the dataset were identified according to the Anatomic Therapeutic Chemical (ATC) classification system (3–7 digits depending upon the medication in question). If necessary, the central pharmaceutical number (PZN, a pharmaceutical product registration code) was used to identify the amount of active ingredient(s) in the prescribed drugs in order to be able to discriminate between PIM drugs and PIM alternatives in cases where discrimination depends upon the concentration of the active substance (e.g. Z-Drugs). Cases (PIM dispensings) and controls (dispensings of PIM alternatives) were defined according to the PRISCUS list. However, as with any other study of claims data, we have no information regarding whether the patient has actually taken the drug or only picked it up in the pharmacy. Hence, patients receiving a PIM / PIM alternative at least once in a quarter were considered the same as patients receiving these drugs more than once per quarter. Since only one of the PIM drugs in the PRISCUS list was in fact new on the market (Prasugrel, a platelet aggregation inhibitor, approval in 2009), we could not apply a new user design to avoid potential confounding by indication. Therefore, we chose an incident user design with a washout period for the study drugs of at least six months preceding the inception (Jan. 1, 2009) of the cohort. This washout period was also applied to hospitalizations in order to avoid the unique situation of two hospital stays during half a year or less.

We defined an exposure-based cohort that started at the time of the first individual dispensing (index date) of PIMs or PIM alternatives. The main outcome was any all-cause hospital admission within 180 days following the index date. Hospitalizations were identified by the presence of a hospital admission day. The day of admission also defines the length of the time interval for the main outcome starting with the index date. We intentionally chose a half year follow-up to avoid counting unexposed cases as exposed. For some patients this time period is too short due to the continued prescription of PIMs for more than half a year, and thus we may have missed hospitalization for the exposed. Nevertheless, we preferred this more conservative approach in order to reduce any potential effect overestimation that might otherwise result.

**Fig 2:**
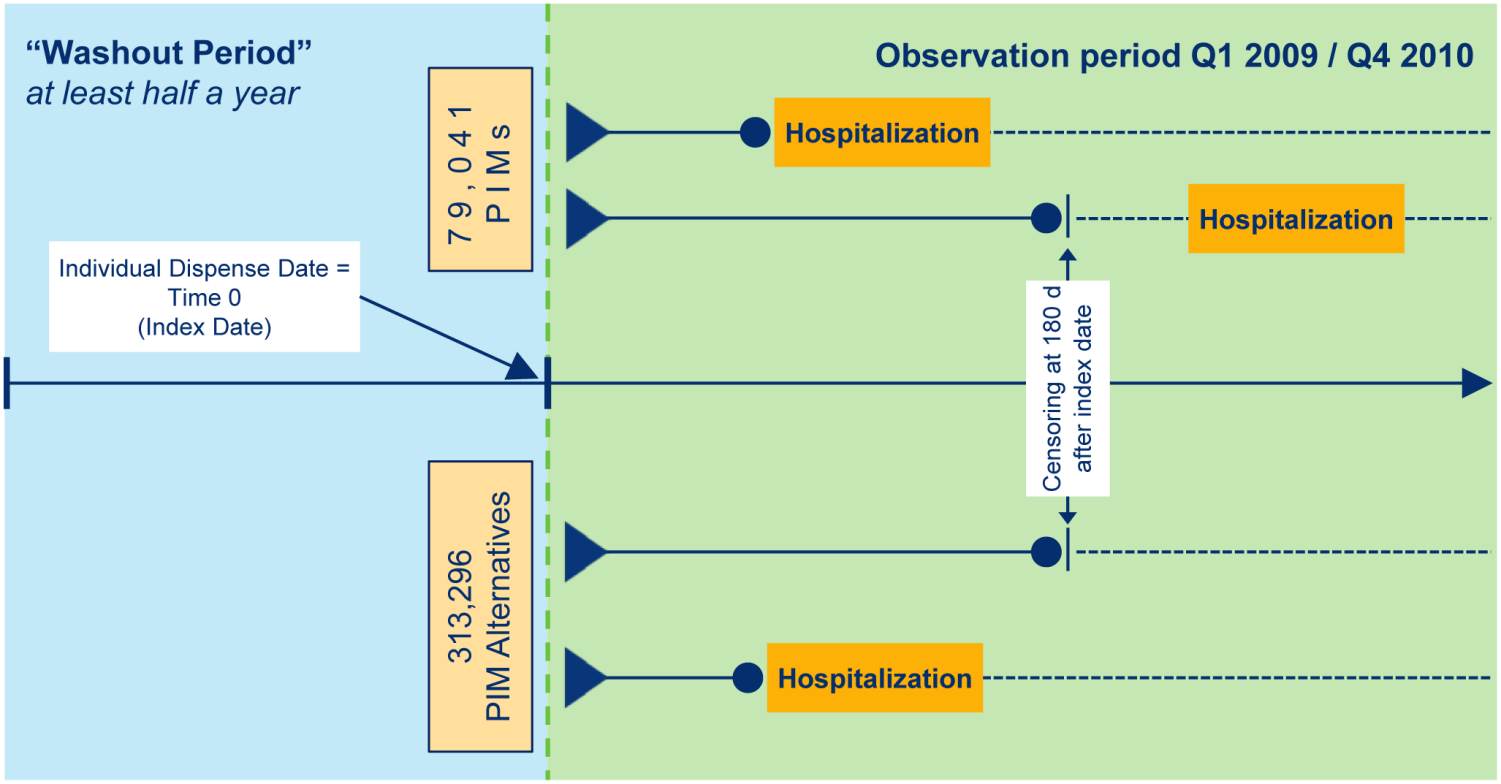
Index date with preceding washout period and subsequent follow-up time. PIM and PIM alternatives according to PRISCUS paper defined by ATC- and PZN-Code. ● End of individual follow-up after dispense date (maximum: 180 days).

If no outcome occurred, follow-up was censored by 180 days (Fig 2). If a hospital admission occurred in the observation period prior to the first PIM dispensing (less than 180 days away from it), the patient was counted as a member of the control group if possible, by taking any PIM alternative dispensing prior to hospitalization as the new index date.

In addition to the main outcome analyses, we used the type of hospitalization (referral by practitioners or emergency hospitalization) for a supplementary analysis, but not the cause of hospitalization which was lacking in the claims data. All confounders were measured in the two quarters before the individual index date of the respective PIM or PIM alternative. Planned sensitivity analysis evaluated the robustness of the main finding by changing the washout period from six months to one year, while maintaining the observation period of 180 days after index date, thus tightening both the definition of incident PIM use and incident hospitalization. In order to extend the washout period from half a year to one year (July 1, 2008 through June 30, 2009), the inception of the cohort was shifted to July 1, 2009.

### Statistics

We used survival analysis to assess the risk of incident all-cause hospitalizations in both patient groups. The time from index date (first pharmacy dispensing of a PIM or PIM alternative) to hospitalization or end of the study after 180 days of follow-up (whichever occurred first) was determined. Kaplan-Meier survival curves were created. A multivariable adjusted Cox proportional hazard model was used to examine the relative risk of all-cause hospitalization within 180 days of initiating PIM / PIM alternative use. For all subjects, all available baseline covariates were extracted from claims data. All analyses were conducted with SAS statistical package (SAS 9.4 for Windows). Kaplan-Meier survival curves were plotted using SPSS 23 (IBM SPSS Statistics).

### Sensitivity analysis

A sensitivity analysis on the length of the washout period was performed. We extended the washout period to one year, i.e. the individual inception of the cohort was not earlier than July 1, 2009. This resulted in a slightly smaller cohort, with 320,012 participants instead of 392,337. Mean age (73.7 years vs. 73.8 years) and median age (73.0 years vs. 73.0 years) remained virtually unchanged, as did the proportion of women (60.4% vs. 60.7%). Maximum age was still 97 years. Both the number of patients with an incident PIM dispensing (56,158) between July 2009 and December 2010, and the number of PIM patients finally belonging to the cases (48,723) decreased equally (39%) and homogeneously over all age groups (65 to 97 years).

### Ethics

In compliance with the German Federal Law on data protection, all data were anonymized to protect the privacy of patients, physicians, and hospitals. Since routine data are pre-existing and anonymized, no ethics committee approval was required.

## Results

The final dataset was a cohort of 392,337 elderly patients, with a total number of 92,243 incident PIM users (23.5% of the cohort). Two-thirds of the PIM users (61,424) were women. For regression analysis the exposed group included 79,041 incident PIM users because the other 13,202 PIM cases had a hospitalization within the 180 days preceding the index date. Hence, the final control group included 313,296 incident users of a PIM alternative according to the PRISCUS list (Fig 1) [7].

We adjusted for all relevant baseline variables, including the implementation of disease management programs (DMPs) yes/no, since DMPs ensure better control of chronic diseases and can consequently significantly lower rates of hospital admissions and readmissions [20]. The variable “long-term care required” (yes/no) is based on an assessment of long-term care needs of the patient by a physician. For long-term care, three different levels of care or care dependency exist. All three levels of care represent a long-term care need by professional nursing staff. The levels of care are available in the claims data. Baseline variables are presented in Table 1.

**Table 1:** Baseline characteristics of older adults classified as cases (PIM group) or controls (PIM alternatives).

Variables*	Cases (%)	Controls (%)	Total (%)	p-Value**	OR***
**Total N**	79,041	313,296	392,337		
**Age (mean / median)**	73.6 / 72.0	73.8 / 73.0	73.8 / 73.0	p < 0.001	
**Male Yes**	25,728 (32.6%)	128,652 (41.1%)	154,380 (39.3%)	p < 0.001	0.693
**No**	53,313 (67.4%)	184,644 (58.9%)	237,957 (60.7%)		
**German**^1^ **Yes**	73,311 (92.8%)	293,960 (93.8%)	367,271 (93.6%)	p < 0.001	0.842
**No**	5,730 (7.2%)	19,336 (6.2%)	25,066 (6.4%)		
**Long-term care required**^2^ **Yes**	6,610 (8.4%)	25,791 (8.2%)	32,401 (8.3%)	p = 0.233	1.017
**No**	72,431 (91.6%)	287,505 (91.8%)	359,936 (91.7%)		
**DMP**^3^ **Diabetes**^4^ **Yes**	14,323 (18.1%)	61,263 (19.6%)	75,586 (19.3%)	p < 0.001	0.911
**No**	64,718 (81.9%)	252,033 (80.4%)	316,751 (80.7%)		
**DMP**^3^ **Lung**^5^ **Yes**	2,960 (3.7%)	9,917 (3.2%)	12,877 (3.3%)	p < 0.001	1.190
**No**	76,081 (96.3%)	303,379 (96.8%)	379,460 (96.7%)		
**DMP**^3^ **Breast Cancer Yes**	225 (0.3%)	713 (0.2%)	938 (0.2%)	p = 0.003	1.252
**No**	78,816 (99.7%)	312,583 (99.8%)	391,399 (99.8%)		
**DMP**^3^ **CHD**^6^ **Yes**	5,777 (7.3%)	25,510 (8.1%)	31,287 (8.0%)	p < 0.001	0.890
**No**	73,264 (92.7%)	287,786 (91.9%)	361,050 (92.0%)		
**CVD**^7^ **Yes**	66,708 (84.4%)	270,566 (86.4%)	337,274 (86.0%)	p < 0.001	0.854
**No**	12,333 (15.6%)	42,730 (13.6%)	55,063 (14.0%)		
**CerebroVD**^8^ **Yes**	12,337 (15.6%)	46,285 (14.8%)	58,622 (14.9%)	p < 0.001	1.067
**No**	66.704 (84.4%)	267,011 (85.2%)	333,715 (85.1%)		
**Cancer Yes**	17,910 (22.7%)	63,661 (20.3%)	81,571 (20.8%)	p < 0.001	1.149
**No**	61,131 (77.3%)	249,635 (79.7%)	310,766 (79.2%)		
**Anemia Yes**	14,920 (18.9%)	60,338 (19.3%)	75,258 (19.2%)	p = 0.015	0.976
**No**	64,121 (81.1%)	252,958 (80.7%)	317,079 (80.8%)		
**Diabetes**^4^ **Yes**	23,127 (29.3%)	95,301 (30.4%)	118,428 (30.2%)	p < 0.001	0.946
**No**	55,914 (70.7%)	217,995 (69.6%)	273,909 (69.8%)		
**Urinary tract**^9^ **Yes**	19,852 (25.1%)	62,092 (19.8%)	81,944 (20.9%)	p < 0.001	1.357
**No**	59,189 (74.9%)	251,204 (80.2%)	310,393 (79.1%)		
**Mean number of ICD10 codes**^10^	16.4 (median 14.0)	13.3 (median 11.0)	13.9 (median 11.5)	p < 0.001	
**Mean number of drug dispensings**^10^	4.8 (median 4.0)	4.2 (median 3.0)	4.3 (median 3.5)	p < 0.001	

^1^ According to identity card,

^2^ Long-term care required (yes/no independent of the extent of required care),

^3^ Participant in Disease Management Program,

^4^ Type I or Type II,

^5^ Asthma or COPD,

^6^ Coronary Heart Disease,

^7^ Cardiovascular Disease,

^8^ Cerebrovascular Disease,

^9^ Kidney and urinary tract,

^10^ in the 6 months preceding index date of PIM or PIM alternative.

All baseline characteristics were assessed during the 6 months preceding the individual index date.

* Variables No = 0 Yes = 1;

** Chi-square or t-test. Multiple testing: the level of significance after Bonferroni adjustment is α = 0.05/16 = 0.003;

*** OR for being in the case group if yes

All baseline characteristics were assessed during the 6 months preceding the individual index date. Women were more often observed in the PIM group (67.4% versus 58.9%). The mean age was almost identical in both groups (73.6 years versus 73.8 years) but with slightly younger ages in the PIM group (median age 72 years versus 73 years). The mean numbers of both, drug dispensings (4.8 versus 4.2) and diagnosed ICD-10 codes (16.4 versus 13.3), were higher in the PIM group. The percentage of patients who need long-term care (8.4% versus 8.2%) and those having an ICD-10 diagnosis of anemia (18.9% versus 19.3%) were quite similar, even though the PIM group had more cancer cases. Across most other baseline characteristics, substantial differences were observed.

On average, each PIM patient had 2.06 PIM dispensings, and 1.35 dispensings per quarter (regardless of the respective drug class). Of all PIM users, 58.4% (53,870) received PIM dispensings in one quarter, 19.3% (17,778) in two quarters, and the remaining 22.3% (20,595) in more than two quarters. The maxima were continuous PIM dispensing over all 8 quarters (660 patients) and up to 14 PIM dispensings in one quarter. Most PIM patients only received a PIM out of one drug class.

The mean number of ICD 10 codes was examined instead of simply summarizing the different codes according comorbidities because the mean number reflects the frequency of visits with ambulatory physicians, both family physicians and medical specialists, during the half year preceding the index date. Recent publications have demonstrated that the frequency of physician visits is reflective of health status, which likewise is an independent risk factor for hospitalization [21]. Analogously, the mean number of drug dispensings in the preceding 6 months before the index date also reflects health status and therefore the risk of being hospitalized. For this calculation, each recorded drug PZN is representative of one dispensed drug (not necessarily with different active ingredients). Since these covariates were extracted for all patients, there were no subjects for whom exposure, confounder, or outcome information was missing.

In addition to Cox regression analysis we calculated the type of admission to hospital, based on the available data, and performed a chi-square test for the comparison of patient groups and type of admission (Table 2). There was no significant difference (p = 0.655) between users of PIM and PIM alternatives.

**Table 2:** Comparison of the type of admission to a hospital in cases (PIM group) and controls (PIM alternatives).

	Cases N (%)	Controls N (%)	Total N (%)
**Elective hospitalization**	7706 (65.3%)	23211 (65.1%)	30917 (65.1%)
**Emergency hospitalization**	4095 (34.7%)	12458 (34.9%)	16553 (34.9%)
**Total**	11801 (100%)	35669 (100%)	47470 (100%)

ChiSq-Test: p = 0.655

We also compared the frequency of polypharmacy in both groups. Polypharmacy was defined as the use of six or more medications and detected by the mean number of PZNs in the two quartiles preceding the index date. This definition is based on the paper of Scott (2015) although one can also find more strict definitions in the literature, e.g. the distinction between minor (2 drugs) and major (>4 drugs) polypharmacy [22, 23]. Unadjusted, polypharmacy was more likely (OR = 1.50; 95% CI: 1.47–1.52; p<0.0001) in the group of patients receiving PIMs compared to the control group (Table 3).

**Table 3:** Polypharmacy in cases (PIM group) and controls (PIM alternatives).

	Cases N (%)	Controls N (%)	Total N (%)
**No Polypharmacy**	62,311 (67.5%)	227,212 (75.7%)	289,523 (73.8%)
**Polypharmacy**	29,932 (32.5%)	72,882 (24.3%)	102,814 (26.2%)
**Total**	92,243 (100%)	300,094 (100%)	392,337 (100%)

Polypharmacy defined as mean number of dispensed drugs >5 in the two quartiles preceding index date

When fully adjusting in a multivariable logistic regression analysis using all variables also used in the Cox regression, polypharmacy was still more likely in the cases (PIM) compared to the controls but with a much smaller OR (OR = 1.187; 95% CI: 1.164–1.210), essentially due to a large influence from the two variables long-term care required (OR = 3.764; 95% CI: 3.665–3.867) and diabetes disease management program (OR = 3.056; 95% CI 3.000–3.112).

In our Cox regression analysis we have adjusted for the mean number of drugs dispensed in the two quartiles preceding the index date, instead of using the binary variable polypharmacy, since the use of a continuous variable is associated with maintaining full information.

**Fig 3:**
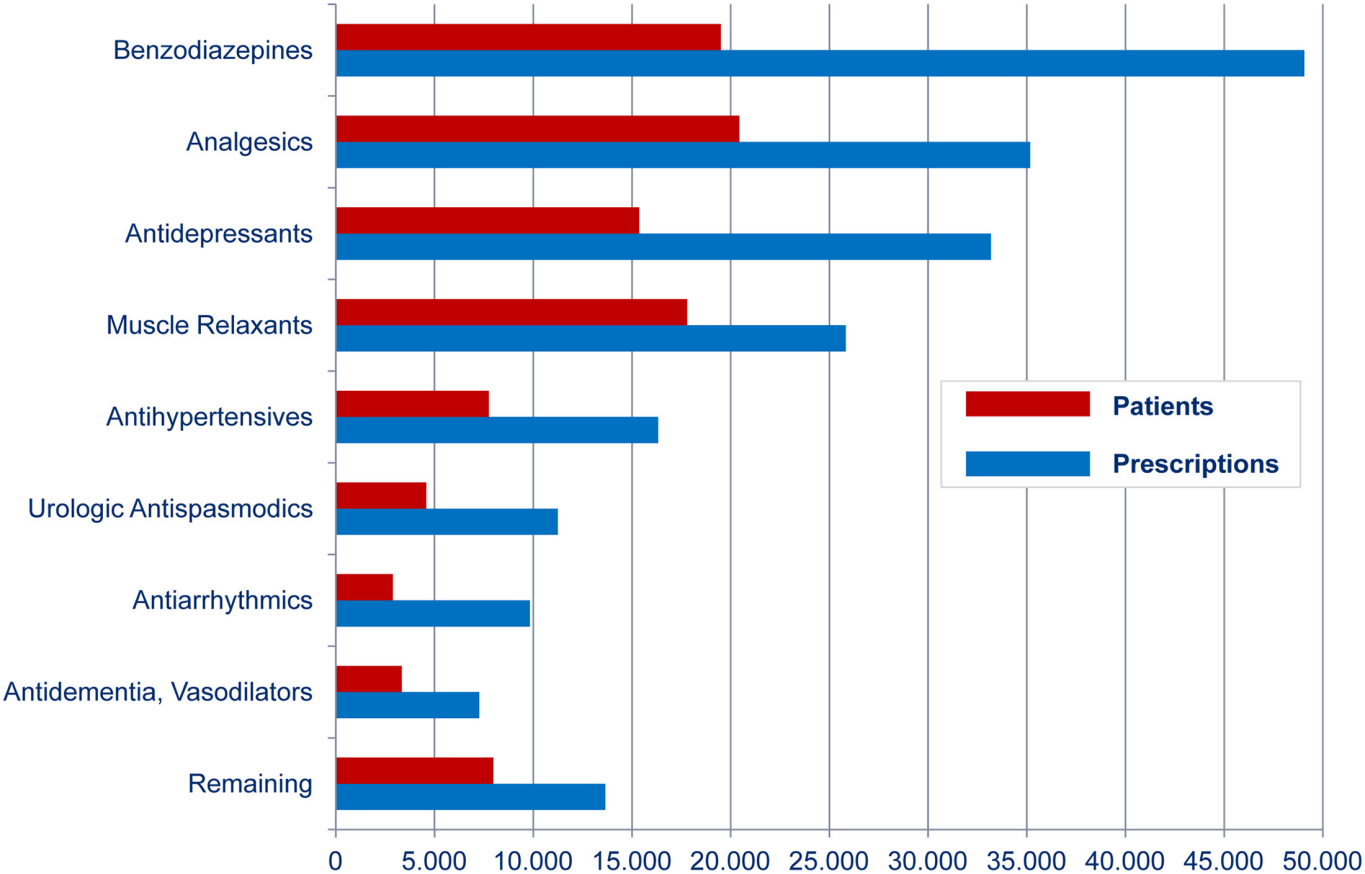
Eight most common PIM drug classes.

The mean number of different PIM drug classes dispensed was 1.26, or, in other words, four out of five PIM patients received a PIM drug from only one drug class, the fifth patient received PIM drugs from two different drug classes. In the latter case, the PIM dispensed first was chosen for Cox regression analysis. Fig 3 summarizes the eight most common PIM drug classes used by the 79,041 PIM patients. The frequency ranking of these top eight drug classes did not differ from the frequency ranking which we found for all 92,243 PIM users. Since we do not have the main diagnosis for the hospital stays in our claims data, we cannot compute statistics regarding the most common diagnoses associated with these eight drugs.

During the entire biennial study period, 126,535 patients (32.3%) of the total study population had an incident hospitalization for any reason. Of these, 79,065 hospitalizations (62.5%) occurred more than 180 days after the index date (PIM group or control group) and therefore were censored. The remaining 47,470 patients were hospitalized for any reason during the 180 days of follow-up (Table 4). Of these patients, 11,801 belonged to the PIM group (14.9% of all patients in this group) and 35,669 to the group taking PIM alternatives (11.4% of all patients in this group). Hospitalization was more likely (OR = 1.37; 95% CI: 1.34–1.40; p<0.0001) in the group of patients receiving PIMs compared to the control group (Table 4).

**Table 4:** Incident hospitalizations during the 180-day follow-up period after the index date in cases (PIM group) and controls (PIM alternatives).

	Cases N (%)	Controls N (%)	Total N (%)
**Hospitalization**	11,801 (14.9%)	35,669 (11.4%)	47,470 (12.1%)
**No Hospitalization**	67,240 (85.1%)	277,627 (88.6%)	344,867 (87.9%)
**Total**	79,041 (100%)	313,296 (100%)	392,337 (100%)

The partially (age and sex) adjusted hazard ratio (HR) exhibited an increased relative risk for all-cause hospitalization in the PIM group (HR: 1.48; 95% CI: 1.45–1.51). After full adjustment for all potential confounders, the relative hospitalization risk (HR: 1.38; 95% CI: 1.35–1.41) remained increased in the PIM group (Table 5). All potential confounders were determined within the 180 days preceding the individual index date.

**Table 5:** Hazard Ratios (HR) for hospitalization within 180 days after index date.

Parameter	HR (95% CI)	p value	HR sensit. (95% CI)	p value
**PIM (1 = yes)**	**1.378 (1.349–1.407)**	**< 0.001**	**1.518 (1.477–1.560)**	**<0.001**
**Age (in 5 year groups)**	1.033 (1.025–1.041)	< 0.001	1.049 (1.039–1.058)	<0.001
**Sex (1 = male)**	1.150 (1.129–1.172)	< 0.001	1.166 (1.141–1.192)	<0.001
**Ethnicity (1 = German)** ^1^	1.033 (0.994–1.074)	0.098	1.006 (0.961–1.053)	0.801
**Care required** ^2^ **(1 = yes)**	1.818 (1.768–1.871)	< 0.001	1.928 (1.864–1.994)	<0.001
**DMP**^3^ **DM**^4^ **(1 = yes)**	0.933 (0.912–0.955)	< 0.001	0.928 (0.903–0.954)	<0.001
**DMP**^3^ **Lung**^5^ **(1 = yes)**	1.163 (1.112–1.216)	< 0.001	1.124 (1.065–1.187)	<0.001
**DMP**^3^ **Breast Cancer (1 = yes)**	2.102 (1.842–2.399)	< 0.001	1.853 (1.558–2.203)	<0.001
**DMP**^3^ **CHD**^6^ **(1 = yes)**	1.331 (1.293–1.371)	< 0.001	1.304 (1.259–1.350)	<0.001
**Urinary tract disease**^7^ **(1 = yes)**	1.049 (1.026–1.072)	< 0.001	1.039 (1.013–1.066)	0.004
**ICD10 mean number** ^8^	1.012 (1.011–1.012)	< 0.001	1.012 (1.011–1.013)	<0.001
**Dispensings mean number** ^8^	1.034 (1.032–1.036)	< 0.001	1.029 (1.026–1.032)	<0.001

^1^ Ethnicity according to identity card (German, other),

^2^ long-term care required irrespective of level of care and facility (residing at home or in a nursing home),

^3^ Disease Management Program,

^4^ Type I or Type II,

^5^ Asthma or COPD,

^6^ Coronary Heart Disease,

^7^ Kidney and urinary tract,

^8^ mean number of ICD10-codings and mean number of drug dispensings (number of drug PZNs) within 6 months before the index date.

**Fig 4:**
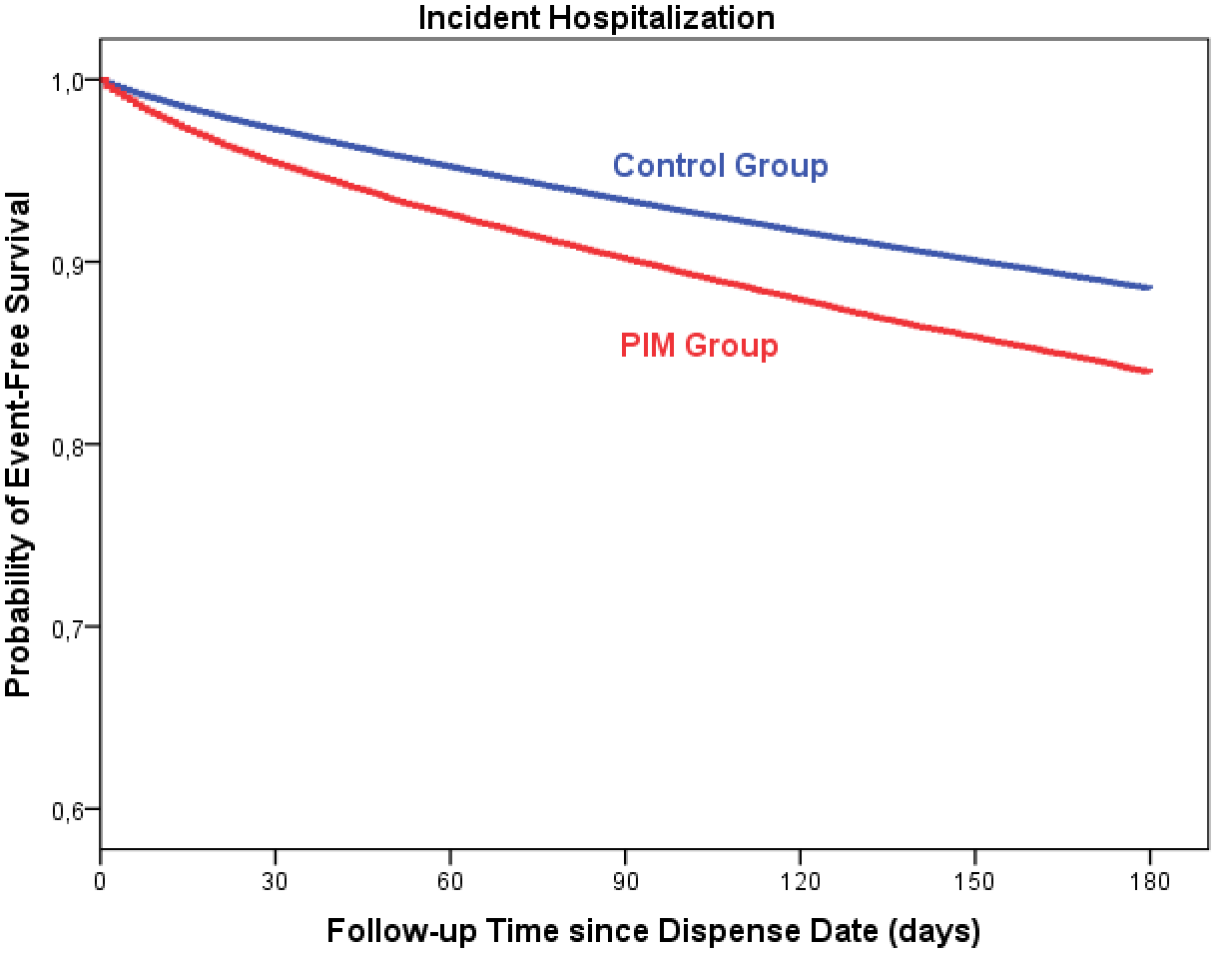
Kaplan-Meier survival curves for hospitalization-free survival during the 180 days of follow-up. Survival curves indicate an association between risk of all-cause hospitalization and use of PIMs.

Fig 4 contains the hospitalization-free survival curves for the first 180 days following index date. The curves reflect the relative risks, with PIM patients generally experiencing more adverse events (hospitalizations) over time.

Expanding the washout period also did not alter the resulting HR substantially. The fully adjusted Cox regression analysis resulted in only a slight increase in all-cause hospitalization risk (HR: 1.51; 95% CI: 1.47–1.56) compared to the hospitalization risk (HR: 1.38) after a wash out period of only half a year (Table 5). The main reason for this slight increase was a larger portion of patients with PIM dispensings exactly covering one or two quarters of the year (85.8% in the sensitivity analysis versus 77.7% in the main analysis), which permitted a better capture of the effective time frame of PIM exposure.

## Discussion

Our results suggest that PIM use among the elderly is widespread and is associated with an increased risk of all-cause hospitalization as compared to PIM alternatives. From nearly 400,000 patients aged 65 years or older in ambulatory care of medical specialists, it was found that 23.5% received pharmacy dispensings of PIMs. Usually, the patients received PIMs for one to two quarters of the year. Thus, PIMs are still taken, at least temporarily, by approximately one-fifth of all elderly patients in ambulatory care. This is consistent with findings from prior studies [18, 24].

PIM use among the elderly is known to be associated with an increased risk of hospitalization through ADEs, drug interactions, or intolerability [25]. Several recently published papers have addressed this and other adverse effects of PIMs [8, 10, 15, 16, 25–27]. However, the heterogeneity of these extensive drug lists (PRISCUS, Beers, and others) poses a challenge when identifying an appropriate control group. It is therefore not surprising that studies often simply compare the group of exposed elderly patients, using any PIM, with a control group of patients not receiving PIMs, but without further differentiation as to whether or not at least one of the drugs used in the control group was considered a PIM alternative [10, 15, 25]. This is less than ideal. We therefore compared PIMs with PIM alternatives as specified in the PRISCUS list. The main outcome was a possible increased hospitalization risk by taking PIMs. We examined possible temporal associations as rigorously as possible, by starting the respective follow-up on the day of the first pharmacy dispensing of a PIM or PIM alternative. As a consequence of the substantially higher prescription rates of PIM alternatives we could not perform a 1:1 comparison of PIMs and PIM alternatives, but we always have used the dispensing date of a PIM alternative as the index date for the control group.

The 79,041 PIM users in our Cox regression model experienced an increased risk of almost 38% for all-cause hospitalization in the first 180 days following the index date. We adjusted for more than 10 different potential confounders, including the number of ICD10 codes and the number of dispensed drugs in the half year before the index date. The latter two reflect the frequency of physician visits during the half year preceding the inception of the cohort and thus the intensity of care and the patient’s health status, which are independent risk factors for hospitalization [21]. If one assumes that the association between PIM use and all-cause hospitalization is causal, then the proportion of hospitalizations associated with PIM use, or, in other words, the population attributable risk percent, is estimated to be 0.059. This means, under the assumption of causality, that almost 6% of the hospitalizations in the entire population of elderly patients could have been avoided if everyone avoided PIMs. We are fully aware of the fact that this is only a theoretical conclusion because we do not know if PIMs are really causal for hospitalization and because PIMs cannot always and everywhere be avoided. A sensitivity analysis in which we extended the washout period to one year resulted in a slight increase of the hospitalization risk (HR = 1.52) in PIM users as compared to controls.

Two recent papers also addressed the question of whether or not the risk of hospitalization increases when PIM drugs defined in the PRISCUS and/or Beers’ list are taken [15, 16]. In particular, the results of Reich, et al. can be compared with ours. In the Cox regression analysis of these Swiss investigators the adjusted HR increased steadily from an HR = 1.13 for patients with one PIM prescription to HR = 1.63 for patients with more than 3 prescriptions., This is probably driven at least in part by the better coverage of the one year follow-up by the increasing number of prescriptions, if one assumes that the one-year observation period was allegedly too long to be always completely covered by PIMs [15]. With the better coverage, a potential exposure misclassification could be avoided. However, due to a lack of clinical information, their study could not adjust for medical diagnoses. A study from Western Australia showed that most of the analyzed PIMs (Beers’ criteria) tended to increase the odds ratios for drug-related hospitalization when the PIM of interest was taken in addition to medications from other drug classes identified as high-risk (e.g. anticoagulants, NSAIDs, corticosteroids, opioids, cardiac rhythm regulators, etc.) [16]. A recently published study from Germany showed that the risk for recurrent falls strongly increased when drugs from the PRISCUS list are dispensed to elderly persons [26], and a very recently published study reported a 46% higher odds of hospitalization for patients aged ≥65 years receiving any PIM compared to those receiving equivalent non-PIMs [27].

In the light of our results, the question arises if PIM alternatives, recommended by the experts involved in the PRISCUS list development [7], are in fact less harmful to older patients. Since we have essentially compared all exposed patients taking any PIM with all unexposed patients taking any PIM alternative, we cannot make any statement regarding the safety of a particular drug or drug class. We only can say that the unexposed group is at a lower risk for subsequent hospitalization and that this is most likely because PIM alternative drugs or drug classes are globally safer than the corresponding PIM drugs.

### Strengths and limitations

Strengths: The routine data used here provide nearly complete information regarding pharmacy dispensing dates, medical status, and health care utilization [25]. Thus, we can rule out any outcome misclassification because the accuracy of the hospital admission date is confirmed for billing reasons. Since we analyzed the routine data of all adults aged ≥65 years, insured by the largest statutory health insurer in this province, we were also able to eliminate selection bias. The association between outcome (hospitalization) and exposure (drug dispensing) is critical. To avoid an exposure misclassification, we restricted the follow-up after first dispensing date to 180 days. Since almost 78% of all patients using PIMs in the main analysis had PIM-dispensings for no more than two quarters of a year, our more conservative approach reflects the reality of exposure better than the one-year observation period used in the study from Switzerland [15]. We thus avoided a potential effect overestimation that might otherwise result.

Limitations: Since we relied on pharmacy dispensing dates to determine drug exposures, we are aware that these data only approximate true daily exposures because actual patient adherence to the medication is unknown, and over-the-counter drugs as well as free samples handed out by physicians are not recorded. Hence, some exposure misclassification may persist. However, pharmacy dispensing dates are considered to be the best possible data source for defining medication exposure in routine settings [28]. Another limitation is the fact that elderly patients have concomitant drugs in addition to PIM or PIM substitutes. We therefore capture in our study the end result of all of these medications. While this may have the advantage of reflecting the real-world experience, if there were an uneven distribution of concomitant drugs in the two patient groups, the HR for all-cause hospitalization might be influenced toward higher or lower risk values. It must also be kept in mind that our routine claims data did not contain anthropometrics or laboratory results, which are often helpful in identifying otherwise unknown comorbidities. Hence, although we found a strong association in our data, one has to consider that a single epidemiologic analysis cannot prove causation.

Our study was designed to examine if the intake of PIMs had any effect on the risk of subsequent hospitalization compared to the use of PIM alternatives. We used a multivariable adjusted Cox proportional hazards regression because this survival analysis, unlike ordinary logistic regression models, takes into consideration time to event and hence handles more effectively the censoring of data. In this way, Cox regression analysis maximizes the use of information on time to event and better estimates the relative risk of hospitalization [29].

As with any nonrandomized study, there is potential for residual confounding. Although we controlled for more than 10 important variables that were very likely to be independent predictors of hospital admissions, the potential for residual confounding remains. Certain known confounders, such as alcohol and tobacco use, use of over-the-counter NSAIDs, immobility, body mass index, and cognitive decline were unmeasured. We tried to estimate health care utilization patterns by analyzing the number of ICD10 codes as a proxy for the number of visits to any physician. Since the incident user design has the disadvantage of depending upon the length of the selected washout period, we performed a sensitivity analysis with a washout period twice as long as in the main analysis. This sensitivity analysis led to a movement of our point estimate (hospitalization risk) away from the null, which supports the validity of the result of the main study.

Since patients and physicians are typically less interested in the metric of an adverse event risk and are more interested in an overall index of benefit or risk, we calculated the population attributable risk percent. With it we can state this risk of hospitalization in a clinically more meaningful metric, albeit with the precondition that there is clear causation. Given that our study did not examine the specific reasons for hospitalizations in the cohort, further studies are needed to better delineate the factors that contribute to this increased hospitalization risk. Nonetheless, our study showed that there is a clear need to monitor elderly patients who use PIMs.

## Conclusions

We compared the safety of PIMs with PIM alternatives, as defined in the PRISCUS list, in a cohort of 392,337 members of a German insurance group who were aged 65 years or older. The clinically relevant adverse event used in the comparison was hospitalization within 180 days following index date. This comparison would likely never be made in the setting of a randomized controlled trial. We adjusted for all available relevant baseline characteristics. HR calculation demonstrated a significant association between PIM dispensing and subsequent excess hospitalization. The population attributable risk percent for PIM users reached almost 6% and, assuming causality, demonstrates the need for monitoring older patients taking PIMs. As a next step, a more specific analysis comparing a single PIM or PIM drug class with the corresponding alternatives could provide a better understanding of the factors that may contribute to the increased hospitalization risk.
